# Supplementary material for: Increased fire activity under high atmospheric oxygen concentrations is compatible with the presence of forests
Source: Nat Commun. 2022 Nov 26;13:7285. doi: 10.1038/s41467-022-35081-z (PMC9701189; doi:10.1038/s41467-022-35081-z)
Supplement: Supplementary file 1 — Supplementary Information [file 41467_2022_35081_MOESM1_ESM.pdf]

Supplementary Information For:

Increased fire activity under high atmospheric oxygen concentrations is compatible with the presence of forests

Rayanne Vitali[1*], Claire M. Belcher[1], Jed Kaplan[2] & Andrew J. Watson[1]

[1]Global Systems Institute, University of Exeter, Exeter, UK

[2]Department of Earth Sciences, The University of Hong Kong, Hong Kong SAR, China

[*] Contact rv237@exeter.ac.uk

Supplementary Methods 1

To model varying Heat of Combustion (HoC) in LPJ-LMfire for given oxygen and PFTs, we first compiled a dataset of HoC values for two given oxygen concentrations (~21% and 100% vol. O_2), shown with sources in Supplementary Table 1. Data was then used to fit Equation 9 for HoC for each PFT in the model (h_{pft}). Where α_{pft} and β_{pft} are PFT coefficients, shown together with R^2 values in Supplementary Table 2.

Supplementary Table 1

Data and sources used to fit Heat of Combustion (HoC) equations for each plant functional type outlined in Supplementary Methods 1, where ambient and bomb values are taken at ~21% and 100% vol. O_2, respectively.

LPJ PFT	Species	Ambient HoC (kJ/g)	Data Source	Bomb HoC (kJ/g)	Data Source
TeNE	Sequioadendron giganteum	10.1	1	20.32	2
	Sequioa sempervirens	11.52	3	20.67	2
	Tsuga heterophylla	11.68	1	19.98697	2
		-	-	20.67942	2
	Abies concolor	10.85	1	21.07	2
	Pinus sylvestris	16.1	4	20.71917	2
		-	-	19.96	2
		-	-	20.23382	2
	Pinus monticola	14.3	4	21.5	2
	Pinus radiata	14.84	3	22.542	2
				21.419	2
TeBE	Podocarpus salignus	15.01	3	22.54	2
	Eucalyptus coccifera	15.17	unpublished*	24	2
	Buxus sp.	18.47	3	21.45	2
TeBS	Acer negundo	14.54	unpublished*	21.037	2
	Alnus rhombifolia	12.08	unpublished*	21	2
	Fraxinus excelsior	12.40	unpublished*	20	2

	Quercus robur	13.30	unpublished*	19.73593	[2]
		-	-	19.82798	[2]
	Quercus cerris	14.49	unpublished*	20.69	[2]
		-	-	20.112	[2]
TrBE	Castanopsis sempervirens	13.52	unpublished*	-	-
	Laurus nobilis	17.38	[3]	20.2	[5]
	Rhobinia neomexicana	13.33	unpublished*	-	-
	machilus odoratissima Nees	-	-	19.84	[6]
	cornus macrophylla wall	-	-	21.74	[6]
TrBR	Acacia auriculiformis	-	-	19.41607	[2]
	Acacia nilotica	-	-	17.66	[2]
	Acacia tortilis	-	-	17.81667	[2]
	Tectona grandis	13.67	[7]	20.3	[8]
	Buchanania obovata	-	-	19.871	[9]
BoNE	Abies picea	15.09	unpublished*	20.06437	[2]
		-	-	20.26207	[2]
	black spruce	14.99	unpublished*	21.038	[2]
BoS	Populus tremuloides	13.1	unpublished*	22	[2]
	Larix occidentalis Nutt.	-	-	18.57	[10]
	Larix occidentalis Nutt.	-	-	20.24	[11]
C4g	Panicum maximum	7.6	[12]	17.677	[13]
		7.4	[12]	-	-
		6.7	[12]	-	-
		7.6	[12]	-	-
	Ctenium concinnum	8.7	[12]	-	-
		8.9	[12]	-	-
		9.4	[12]	-	-
		9.2	[12]	-	-
	Trachypogon spicatus	8.2	[12]	-	-
		9	[12]	-	-
		8.5	[12]	-	-
	Melinis repens	8.20	[12]	-	-
		8.40	[12]	-	-
		8.60	[12]	-	-
		7.90	[12]	-	-
	Pennisetum	7.9	[12]	-	-
		7.6	[12]	-	-
		7.5	[12]	-	-

	7.9	12	-	-
Sporobolus pyramidalis	8	12	-	-
	7.5	12	-	-
	8.2	12	-	-
	8.1	12	-	-
Aristida rufescens	9.1	12	-	-
	9.3	12	-	-
	8.4	12	-	-
	8.3	12	-	-
Digitaria longiflora	9	12	-	-
	9.4	12	-	-
	9	12	-	-
	9.4	12	-	-
Aristida congesta var barbicolis	8.45	14	-	-
	8.7	14	-	-
	8.5	14	-	-
Alloteropsis semialata	9.3	14	-	-
	10	14	-	-
	8.4	14	-	-
Melinis sp	10.2	14	-	-
	9.2	14	-	-
	10.8	14	-	-
Merxmuellera stricta	10.7	14	-	-
	9.6	14	-	-
	11.85	14	-	-
Cynodon dactylon	11.3	14	-	-
	11.55	14	-	-
	11.2	14	-	-
Cymbopogon nardus	10.15	14	-	-
	8.8	14	-	-
	10.7	14	-	-
Digitaria eriantha	8.5	14	17.538	13
	9.05	14	17.538	13
	8.466667	14	17.538	13
Eragrostis curvula	9.4	14	-	-
	8.25	14	-	-
	9.05	14	-	-
Eragrostis lehmanniana	8.733333	14	-	-

	9	14	-	-
	8.75	14	-	-
Pentameris sp	10.25	14	-	-
	12.1	14	-	-
	12	14	-	-
Eragrostis plana	10.00	14	-	-
	9.95	14	-	-
	10.45	14	-	-
Eustachys paspaloides	6.45	14	-	-
	8.4	14	-	-
	8.25	14	-	-
Heteropogon contortus	11.45	14	-	-
	10.55	14	-	-
	10.85	14	-	-
Hyparrhenia hirta	8.2	14	-	-
	8.85	14	-	-
	7.45	14	-	-
Melica racemosa	9.95	14	-	-
	9.7	14	-	-
	11.25	14	-	-
Panicum aequinerve	10.2	14	-	-
	9.3	14	-	-
	9.2	14	-	-
Panicum sp	8.95	14	-	-
	10.15	14	-	-
	8.333333	14	-	-
Cenchrus setaceus	7.35	14	-	-
	7.5	14	-	-
	6.9	14	-	-
Melinis nerviglumis	12.45	14	-	-
	12.70	14	-	-
	12.07	14	-	-
Setaria sphacelata	7.967	14	-	-
Sporobolus africanus	11.6	14	-	-
	10.75	14	-	-
	11.1	14	-	-
Themeda triandra	8.9	14	17.727	13
	8.85	14	17.727	13

		8.75	14	17.727	13
	Sporobolus fimbriatu	-	-	17.212	13
C3g	Cymbopogon plurinodis	-	-	18.133	13
	Rubus fruticosus	14.63	3	-	-
	Urtica dioica	13.26	3	20.87	2
	Piper nigrum	14.04	3	-	-
	Sacandra chloranthoides	15.79	3	-	-

*Unpublished data collected by C. M. Belcher following methods presented in [3]

Supplementary Table 2

PFT parameters used in the model where $ieff_{pft}$ is ignition efficiency for each PFT, whilst α_{pft} and β_{pft} are fitted parameters used to fit equation for each PFT outlined in Supplementary Methods 1 together with R^2 statistics. With TrBE = tropical broadleaf evergreen, TrBR = tropical broadleaf raingreen, TeNE = temperate needleleaf evergreen, TeBE = temperate broadleaf evergreen, TeBS = temperate broadleaf summergreen, BoNE = boreal needleleaf evergreen, BoS = boreal summergreen, C3gr =C3 perennial grass and C4gr = C4 perennial grass.

	TrBE	TrBR	TeNE	TeBE	TeBS	BoNE	BoS	C3	C4
$ieff_{pft}$	0.05	0.40	0.10	0.10	0.50	0.44	0.44	0.5	0.5
α_{pft}	-155.03	-144.59	-190.4	-170.85	-185.05	-143.06	-190.02	-134.39	-221.5
β_{pft}	22.14	20.43	22.73	24.34	22.19	21.89	22.17	20.85	19.8
R^2	0.8	0.8	0.83	0.85	0.95	0.99	0.87	0.83	0.74

Supplementary Table 3

Datasets used to drive LPJ-LMfire

Variables	Data sets	References
Baseline climatology: Long-term monthly means temperature, precipitation, diurnal temperature range	WorldClim 2.1; Climate WNA	15,16
number of days per month with precipitation, wind speed	CRU CL 2.0	17
total cloud cover	Wisconsin HIRS Cloud Climatology	18
lightning flashes	LIS/OTD HRMC	19
Climate interannual variability: Detrended and transient (1871–2010) temperature, precipitation, cloud cover, wind speed, CAPE	20th Century Reanalysis	20
Elevation and Slope	ETOPO1	21
Soil particle size distribution and volume fraction of coarse fragments	Harmonized World Soil Database	22
Atmospheric CO2 concentrations	Composite CO2 time series	23

Supplementary References

1. Belcher, C. M. The influence of leaf morphology on litter flammability and its utility for interpreting palaeofire. *Philosophical Transactions of the Royal Society B: Biological Sciences* **371**, (2016).
2. Rivera, J. de D., Davies, G. M. & Jahn, W. Flammability and the heat of combustion of natural fuels: a review. *Combustion science and technology* **184**, 224–242 (2012).
3. Belcher, C. M. & Hudspith, V. A. Changes to Cretaceous surface fire behaviour influenced the spread of the early angiosperms. *New Phytologist* **213**, 1521–1532 (2017).
4. Dewhirst, R. A., Smirnoff, N. & Belcher, C. M. Pine species that support crown fire regimes have lower leaf-level terpene contents than those native to surface fire regimes. *Fire* **3**, 17 (2020).
5. Nunez-Regueira, L., Rodríguez-Añón, J. A., Proupín-Castiñeiras, J. & Nunez-Fernandez, O. Calculation of forest biomass indices as a tool to fight forest fires. *Thermochimica Acta* **378**, 9–25 (2001).
6. Jain, R. K. Fuelwood characteristics of some tropical trees of India. *Biomass and Bioenergy* **4**, 461–464 (1993).
7. White, R. H., Dietenberger, M. A. & Stark, N. M. Cone calorimeter tests of wood-based decking materials. in *Proceedings of the 18th annual conference on recent advances in flame retardancy of polymeric materials.* 326–337 (2007).
8. Günther, B., Gebauer, K., Barkowski, R., Rosenthal, M. & Bues, C.-T. Calorific value of selected wood species and wood products. *European Journal of Wood and Wood Products* **70**, 755–757 (2012).
9. Bowman, D. & Wilson, B. A. Fuel characteristics of coastal monsoon forests, Northern Territory, Australia. *Journal of Biogeography* **15**, 807–817 (1988).
10. Gower, S. T., Grier, C. C. & Vogt, K. A. Aboveground production and N and P use by Larix occidentalis and Pinus contorta in the Washington Cascades, USA. *Tree physiology* **5**, 1–11 (1989).
11. Kelsey, R. G. *Heat Content of Bark, Twigs, and Foliage of Nine Species of Western Conifers*. vol. 261 (Forest Service, US Department of Agriculture, Intermountain Forest and Range …, 1979).
12. Solofondranohatra, C. L. *et al.* Shade alters the growth and architecture of tropical grasses by reducing root biomass. *Biotropica* **53**, 1052–1062 (2021).
13. Trollope, W. S. W. Control of Bush Encroachment with Fire in the Arid Savannas of Southeastern Africa. (University of Natal, 1983).
14. Simpson, K. J. *et al.* Determinants of flammability in savanna grass species. *Journal of Ecology* **104**, 138–148 (2016).
15. Wang, T., Hamann, A., Spittlehouse, D. L. & Murdock, T. Q. ClimateWNA—high-resolution spatial climate data for western North America. *Journal of Applied Meteorology and Climatology* **51**, 16–29 (2012).

16. Hijmans, R. J., Cameron, S. E., Parra, J. L., Jones, P. G. & Jarvis, A. Very high resolution interpolated climate surfaces for global land areas. *International Journal of Climatology: A Journal of the Royal Meteorological Society* **25**, 1965–1978 (2005).
17. New, M., Lister, D., Hulme, M. & Makin, I. A high-resolution data set of surface climate over global land areas. *Climate research* **21**, 1–25 (2002).
18. Wylie, D., Jackson, D. L., Menzel, W. P. & Bates, J. J. Trends in global cloud cover in two decades of HIRS observations. *Journal of climate* **18**, 3021–3031 (2005).
19. Christian, H. J. *et al.* Global frequency and distribution of lightning as observed from space by the Optical Transient Detector. *Journal of Geophysical Research: Atmospheres* **108**, ACL 4-1-ACL 4-15 (2003).
20. Compo, G. P. *et al. The Twentieth Century Reanalysis Project, Qj Roy. Meteor. Soc., 137, 1–28*. (2011).
21. Amante, C. & Eakins, B. W. *Etopo1 Arc-Minute Global Relief Model: Procedures, Data Sources and Analysis*. (US Department of Commerce, National Oceanic and Atmospheric Administration, National Environmental Satellite, Data, and Information Service, National Geophysical Data Center, Marine Geology and Geophysics Division, 2009).
22. Batjes, N. H. *ISRIC-WISE Harmonized Global Soil Profile Dataset*. (ISRIC-World Soil Information, Wageningen, The Netherlands, 2008).
23. Krumhardt, K. M. & Kaplan, J. O. *A spline fit to atmospheric CO2 records from Antarctic ice cores and measured concentrations for the last 25000 years*. (2012).
